# Distribution and antibiogram of *Vibrio* species from hospital wastewater in Southwest, Nigeria

**DOI:** 10.11604/pamj.2023.45.80.35773

**Published:** 2023-06-09

**Authors:** Temitope Deborah Agboola, Eucharia Ezenwanyi Nmema, Babatunde Wumi Odetoyin

**Affiliations:** 1 Department of Biological Sciences, Olusegun Agagu University of Science and Technology, Okitipupa, Ondo State, Nigeria,; 2 Department of Medical Microbiology and Parasitology, Obafemi Awolowo University, Osun State, Nigeria

**Keywords:** Wastewater, hospital, *Vibrio cholerae*, polymerase chain reaction, sequencing

## Abstract

**Introduction:**

the continuous generation of wastewater and its release into the environment with little or no treatment remains a threat to the environment and public health. We examined the prevalence and antibiotic susceptibility profiles of Vibrio species isolated from untreated wastewater samples from Ondo State Specialist Hospital Okitipupa, Nigeria, as part of the global efforts to provide information for containing the spread of resistant infections.

**Methods:**

twelve hospital wastewater samples were collected aseptically and transported to the laboratory for analysis. The samples were processed on thiosulphate citrate bile salt sucrose agar and colonies typical of Vibrio species were selected for further identification. All isolates were confirmed by polymerase chain reaction (PCR) using Vibrio-specific primers and the PCR products were sequenced for species identification. The susceptibility profiles of the isolates were determined by the Kirby-Bauer disc diffusion method.

**Results:**

twenty-nine (58%) of 38 presumptive isolates were confirmed as Vibrio by PCR, while 23 (60.5%) isolates were screened up to species level by sequencing. Six different species following the trend: 26.1% V. fortis and V. algivorus, 17.4% V. cholerae, 13.0% V. panuliri, 8.7% V. stylophorae and V. parahaemolyticus were identified. The isolates were commonly resistant (73.9%-91.3%) to doxycycline, tetracycline, erythromycin and meropenem. The least resistance rate (17.4%) was observed against amikacin and cotrimoxazole. All isolates were multidrug-resistant, with multiple antibiotic resistance (MAR) indices exceeding the 0.2 recommended limit.

**Conclusion:**

this study has shown that untreated hospital wastewater is a reservoir for diverse strains of multiply resistant Vibrio species. Therefore, it is essential to adequately treat hospital wastewater to eliminate these emerging pollutants and set up a monitoring scheme to evaluate the treatment plants' effectiveness to reduce the pollutants' impact on the environment and the population.

## Introduction

Wastewater generation is a phenomenon that is common to the whole world and treatment of wastewater is imperatively demanded by authorities before it is discharged into the environment [1]. Wastewater facility´s obligation is to provide quality ways of managing their wastewater effluents before its release into the environment but most of these facilities still release final effluents that contain certain amounts of pathogenic bacteria [2]. The release of wastewater with contaminants such as pathogens into the environment threatens the environmental biota as well as water bodies needed in society [3]. Increased pollution of the environment via the discharge of effluents from diverse sources including hospitals had resulted in many water-borne infectious disease epidemics in both developing and some developed countries. Wastewater could be composed of physical (odour, colour), chemical (toxic organic substances, alkaline), and biological materials (pathogens, animals) which is a function of the source of the wastewater [4]. The extent to which wastewater affects the environment is closely linked to its constituent. The difference in wastewater is influenced by certain parameters such as population, natural environment, and recreational, domestic, and industrial-related activities around the area [5]. Most of the activities carried out in the hospital include finding solutions to one infectious disease or the other which could also be shed via fecal content into the hospital sewage system. Along with excreta, they flow with other wastewater to the sewage treatment plant. The final effluent may still contain bacteria such as *Vibrio* and be discharged into neighboring aquatic bodies after passing through the sewage system [1]. *Vibrio* species (common pollutants in contaminated or wastewater) are gram-negative, non-spore formers, rod-shaped with a straight single rigid curve, and motile bacteria possessing single polar flagellum when cultured in broth [6]. These organisms are natural constituents of diverse aquatic environments. About 130 species had been documented meanwhile about 12 species such as *V. cholerae, V. parahaemolyticus, V. vulnificus, V. fluvialis, V. alginolyticus, V. harveyi*, V. *damsela, V. hollisae, V. cincinnatiensis, V. furnissii, V. metschnikovii*, and *V. mimicus*, are recognized to be pathogenic to human [7]. Several studies had been conducted on the incidence of *Vibrio cholerae* and *V. parahaemolyticus*, meanwhile some other species of *Vibrio* known to be of medical interest termed emerging pathogens could also be associated with a mild or severe infection in humans [3,8]. The species of this pathogen that are of public health importance and known to be transferred via water and aquatic animals include *V. parahaemolyticus, V. vulnificus, V. fluvialis, V. tubiashi*, and *V. cholerae* [9].

Globally, reports show that about 3-5 million individuals contract cholera yearly which is accompanied by about 100,000 deaths, especially in endemic areas of the world. Children below five years in age are said to be involved in half of the deaths reported [10]. Children in this age group experience the highest incidence of cholera with variation in the incidence annually which could be as a result of some factors including immune status and climatic factors. *V. cholerae* comprises more than two hundred serotypes which are differentiated based on the composition of O- antigen of lipopolysaccharide chemically and this serogroup O1 is reported to cause most cholera cases meanwhile, the O139 strain is said to be found sporadically and not linked with any major disease outbreak [11]. Non- O1 and non- O139 *V. cholerae* serotypes which are understudied *Vibrio* strains compared with O1 and O139 strains pathogenic to humans had been linked to sporadic and severe, gastrointestinal and extraintestinal infections [12]. *V. parahaemolyticus* is another common *Vibrio* species, with sporadic cases of infection in coastal areas, often associated with the consumption of raw or minimally processed contaminated seafood, particularly during the warmer months, [12] although infection could also occur as a result of wound exposure to polluted water. *V. parahaemolyticus* variant of the serotype O3: K6 had been reported to be associated with some outbreaks and this strain possesses certain virulent genes such as direct hemolysin encoded by *tdh* [12,13]. *V. vulnificus* is an opportunistic pathogen that is often found in estuarine waters and has been recovered from a variety of environmental sources, including sea creatures, sediment, and seawater [14]. Most infections caused by *V. vulnificus* have a link with an underlying illness like cirrhosis or hepatitis, diabetes mellitus, and malignancies, and these infections have fatality rates ranging from 30 to 50% [15]. *Vibrio vulnificus* had been recovered from soft tissue wounds caused by fish fins and several studies revealed that accumulation of *V. vulnificus* in fish is associated with its presence in freshwater which could be as a result of water body´s contamination due to inflow of wastewater from diverse sources [16]. This is due to their involvement in seafood-associated or foodborne disease outbreaks in several parts of the world, including Europe, the United States, and Malaysia [17,18]. Wastewater treatment plant effluents had been reported as an important source of bacteria such as *Vibrio* as well as antibiotic-resistance genes released into the downstream environment [19]. Waterborne diseases continually threaten public health globally due to the discharge of waste from different sources (including hospitals) into water bodies even though there are major improvements in water quality as well as treatment of wastewater [3]. Hospital wastewater also contains an appreciable number of antibiotic-resistant pathogens which could be introduced into the sewage system and when discharged into nearby water bodies compound the incidence of waterborne infectious diseases when such water is consumed directly or indirectly [20]. Hence, this study assessed the distribution of *Vibrio* species in wastewater of State Specialist Hospital Okitipupa, Ondo State, Nigeria as well as the antibiotic susceptibility profiles of the recovered isolates.

## Methods

**Description of study site:** this study was conducted in Okitipupa, Ondo State, Southwest Nigeria. The coordinates of the sample sites within the hospital are N 6^o^ 44´ 85.7´´, E 4^o^ 77´ 36.1´´, N 6^o^ 49´77.4´´, E 4^o^ 78´60.2´´, and N6^o^ 50´57.1´´ E 4^o^ 78´ 50.4´´. The septic tank is the main means of collecting wastewater in the hospital and usually undergoes minimal or no treatment before it is discharged into nearby water bodies or buried underground.

**Sample collection and isolation of *Vibrio* species:** wastewater samples were collected aseptically using sterile 1L sample bottles from three hospital wards (male ward, female ward, and outpatient department) and transported on ice from the collection site to the laboratory for analysis. All samples were analyzed within 12 h of sample collection. Wastewater samples were enriched in alkaline peptone water, incubated at 37°C overnight, and then diluted. About 0.1 ml of the diluent was inoculated on sterile thiosulphate citrate bile salts sucrose (TCBS) (Himedia, India) agar using the spread plate method and incubated at 37°C for 48 h. Two to five colonies that showed the features of *Vibrio* on TCBS plates were picked randomly and subsequently purified on sterile nutrient agar plates. The pure isolates were Gram-stained and an oxidase test was performed. Only the Gram-negative, oxidase-positive isolates were selected for further confirmation.

**Extraction of DNA:** the DNA of the isolates was extracted as described by Adesiyan *et al*. [21]. Single colonies of presumptive isolates of *Vibrio* cultured overnight on nutrient agar plates at 37°C were picked and suspended in 100 µL of sterile distilled water. The cells were lysed using Dri-Block (Techne, USA) at 100°C for 15 min, allowed to cool, and centrifuged at 13000 rpm for 15 min using a Biofuge A micro centrifuge (Heraeus Sepatech GmbH, Germany). The cell lysates were used as DNA templates in the PCR assays.

**Molecular identification of *Vibrio* species:** identification of the genus of the *Vibrio* isolates was carried out using a PCR-based method with the primer sequence F: CGGTGAAATGCGTAGAGAT, R: TACTAGCGATTCCGAGTTC. The total reaction mixture was 25 μL which consisted of 12.5μL 2X Master Mix with standard buffer (Biolabs, United Kingdom), 1μL of each primer (Inqaba Biotech, South Africa), 5.5μL of nuclease free water (Amresco, United States), and 5μL of template DNA. *Vibrio parahaemolyticus* DSM 10027 and sterile distilled water were used as positive and negative controls respectively. Amplification conditions were initial denaturation at 93°C for 15 min followed by 35 cycles of 92 °C for 40 s, annealing: 45°C for 1 min, elongation at 72 °C for 1.5 min, and final elongation at 72 °C for 7 min. The amplicons were sent for partial sequencing using the Sanger method for species identification.

**Antibiotic susceptibility testing of *Vibrio* isolates:** the antibiotic susceptibility test was carried out by employing the standard disc diffusion method on Mueller-Hinton agar (MH) as described by Bauer [22]. Fifteen antibiotics including those recommended by the Centre for Disease Control and Prevention (CDC) as well as those commonly used for the treatment of diarrhoeal associated infections were selected as follows: gentamicin (10 μg), amikacin (30 μg), streptomycin (300 μg), cefotaxime (30 µg), meropenem (10µg), chloramphenicol (30 µg), ciprofloxacin (5µg), ampicillin (10µg), sulfamethoxazole (25µg) tetracycline (30 µg), trimethoprim + sulfamethoxazole (25 µg), erythromycin (15 µg), amoxycillin (25 µg), doxycycline (30 µg) and rifampicin (5 µg). The results were recorded as susceptible or resistant using the Clinical Laboratory and Standard institute (CLSI) zone diameter interpretation guidelines [23].

**Multiple antibiotic resistant phenotypes and index:** the frequencies, percentages, antibiotic resistance profile, and multiple antibiotic resistance phenotypes (MARPs) of *Vibrio* species were determined, and isolates showing resistance to more than two classes of antibiotics were recorded. The multiple antibiotic resistance (MAR) index is an important indicator used to identify the risk source of contamination with potential hazards to humans [24]. MAR index = a/b, where a = total antibiotics to which resistance was recorded, and b = total antibiotics to which each isolate was exposed. Also, the antibiotic resistance pattern abundance (ARPA) was calculated using the following formula. Resistance pattern abundance= RT/TS, where RT is the number of resistance types and TS is the total number of strains assayed [25].

**Data analysis** was done using Excel software package (2016). Data were presented in a tabular form as percentages and frequencies.

## Results

**Figure 1:**
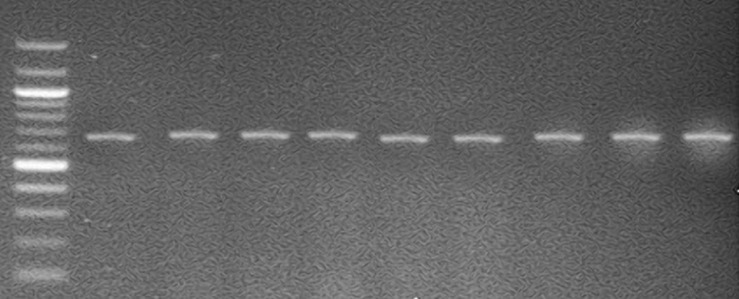
gel electrophoresis of genus *Vibrio*

**Prevalence and distribution of *Vibrio* species:** thirty-eight presumptive *Vibrio* species were recovered after Gram staining and oxidase confirmatory test. However, 29 (58%) isolates were confirmed by PCR while 23 (60.5%) isolates were screened up to species level by sequencing (Figure 1). The sequence results revealed six different species following the trend: 26.1% *V. fortis* and *V. algivorus*, 17.4% *V. cholerae*, 13.0% *V. panuliri*, 8.7% *V. stylophorae* and *V. parahaemolyticus* (Table 1). Figure 1.

**Table 1:** distribution of *Vibrio* species

Isolate code	Query cover	Percentage identity	Accession number	Identity
V1	99	93.73	NR_025575.1	*Vibrio fortis*
V11	97	90.41	NR_025575.1	*Vibrio fortis*
V17	98	90.85	NR_025575.1	*Vibrio fortis*
V12	97	90.41	NR_025575.1	*Vibrio fortis*
V31	95	88.74	NR_025575.1	*Vibrio fortis*
V19	93	92.35	NR_025575.1	*Vibrio fortis*
V2	91	93.08	MF772495.1	*V. cholerae*
V8	96	90.86	MF772495.1	*V. cholerae*
V7	97	95.17	KT270307.1	*V. cholerae*
V25	98	98.54	KT270307.1	*V. cholerae*
V3	60	74.60	FN997623.1	*V. parahaemolyticus*
V5	45	74.71	KM505084.1	*V. parahaemolyticus*
V27	97	92.49	NR_151933.1	*V. algivorus*
V20	97	92.97	NR_151933.1	*V. algivorus*
V14	76	92.60	NR_151933.1	*V. algivorus*
V33	99	93.41	NR_151933.1	*V. algivorus*
V16	89	90.62	NR_151933.1	*V.algivorus*
V29	92	73.89	NR_151933.1	*V.algivorus*
V6	99	91.23	NR_136876.1	*V.algivorus*
V21	99	91.37	NR_136876.1	*V. algivorus*
V15	96	93.45	NR_136876.1	*V. algivorus*
V35	60	87.66	NR_108575.1	*V. stylophorae*
V4	82	91. 35	NR_108575.1	*V. stylophorae*

**Antibiotic Susceptibility profiles of *Vibrio* isolates:** as shown in Table 2, the isolates were commonly resistant to erythromycin (91.3%), doxycycline (82.6%), meropenem (78.3%), tetracycline (73.9%) and cefotaxime (69.6%). The least resistance rate was observed against amikacin (17.4%) and cotrimoxazole (17.4%). All the *Vibrio cholerae* strains exhibited high rates of resistance (75-100%) to cefotaxime, erythromycin, doxycycline and tetracycline. They were however 100% sensitive to gentamicin and cotrimoxazole. *Vibrio parahaemolyticus* isolates were 100% susceptible to amikacin, ciprofloxacin, cotrimoxazole and chloramphenicol while they were 100% resistant to tetracycline and erythromycin. *V. algivorus* showed resistance to the antibiotics tested ranging from 33.3 - 83% with the highest percentage observed against cefotaxime, doxycycline, and erythromycin. *V. panuliri* was 100% susceptible to amikacin, ciprofloxacin, cotrimoxazole, and chloramphenicol while they were 100% resistant to cefotaxime, meropenem, rifampicin, and erythromycin. *V. fortis* was 100% resistant to the tetracycline class of antibiotics while susceptibility to other antibiotics ranges between 16.7 - 83%. *V. stylophorae* was 100% susceptible to Amikacin and six other antibiotics but showed 100% resistance to meropenem, doxycycline, and erythromycin.

**Table 2:** percentages of antibiotic-resistant *Vibrio* species (N=23) from hospital wastewater in Okitipupa

Antibiotic class	Antibiotic		*Vibrio cholerae* (n = 4)	*Vibrio panuliri* (n = 2)	*Vibrio algivorus* (n = 6)	*Vibrio fortis* (n = 6)	*Vibrio parahaemolyticus* (n = 3)	*V. stylophorae* (n = 2)	Total (n=23)
		P (μg)	R (%)	R (%)	R (%)	R (%)	R (%)	R (%)	R (%)
Aminoglycosides	Amikacin	30	1 (25)	0 (0)	2 (33.3)	1 (16.7)	0 (0)	0 (0)	4 (17.4)
	Gentamicin	10	0 (0)	1 (50)	3 (50)	3 (50)	1 (33.3)	0 (0)	8 (34.8)
	Streptomycin	10	2 (50)	1 (50)	2 (33.3)	2 (33.3)	1 (33.3)	1 (50)	9 (39.1)
Fluoroquinolone	Ciprofloxacin	5	1 (25)	0 (0)	4 (66.7)	1 (16.7)	0 (0)	0 (0)	6(26.1)
Sulphonamides	Cotrimoxazole	25	0 (0)	0 (0)	2 (33.3)	2 (33.3)	0 (0)	0 (0)	4(17.4)
	Trimethoprim	25	2 (50)	1 (0)	3 (50)	3 (50)	1 (33.3)	1 (50)	11 (47.8)
Carbapenems	Meropenem	10	3 (75)	2 (100)	4 (66.7)	5 (83)	2 (66.7)	2 (100)	18(78.3)
Cephalosporin	Cefotaxime	30	4 (100)	2 (100)	5 (83)	3 (50)	1 (33.3)	1 (50)	16(69.6)
Phenicol	Chloramphenicol	30	2 (50)	0 (0)	3 (50)	2 (33.3)	0 (0)	0 (0)	7(30.4)
Rifamycin	Rifamycin	5	4 (100)	2 (100)	4 (66.7)	5 (83)	2 (66.7)	1 (50)	18(78.3)
Tetracycline	Tetracycline	30	3 (75)	1 (50)	4 (66.7)	6 (100)	3 (100)	0 (0)	17(73.9)
	Doxycyline	30	3 (75)	2 (100)	5 (83)	6 (100)	1 (33.3)	2 (100)	19(82.6)
Macrolides	Erythromycin	5	4 (100)	2 (100)	5 (83)	5 (66.7)	3 (100)	2 (100)	21(91.3)
β-lactams	Ampicillin	10	1 (25)	0 (0)	2 (33.3)	2 (33.3)	1 (33.3)	0 (0)	6 (26.1)
	Amoxycillin	25	2 (25)	0 (0)	2 (33.3)	3 (50)	2 (66.7)	0 (0)	9 (39.1)

**Multiple Antibiotic Resistance Phenotypes of *Vibrio* species:** all the species of *Vibrio* isolated in this study showed resistance to more than two classes of antibiotics which indicates multiple antibiotic resistance. Hence, the multiple antibiotic resistance phenotypes were assessed and the results showed that the resistance phenotype ranges between 4 - 9 classes of antibiotics (Table 3). Resistance to four classes of antibiotics was found in *V. algivorus* and *V. stylophorae* which was the lowest incidence while two strains of *Vibrio algivorus* were resistant to nine classes of antibiotics, the highest incidence of multiple antibiotic resistance. The MAR indices of all the isolates were higher than the 0.2 recommended threshold. *V. cholerae* had MAR indices ranging from 0.4 - 0.6, *V. parahaemolyticus* was 0.5, *V. algivorus* had values ranging from 0.3 - 0.7, *V. fortis* had 0.5 - 0.6 MAR indices while *V. panuliri* had 0.3 - 0.5 and *V. stylophorae* had indices that ranged from 0.3 - 0.4.

**Table 3:** multiple antibiotic resistance phenotypes patterns and index of the *Vibrio* pathotypes

Classes of antibiotics	Phenotypes	Frequency of occurrence	Multiple antibiotic resistance index	Antibiotic resistance pattern abundance
	*V. cholerae*			
5	Ceph-Rif-Tet-Mac-Lac	1	0.4	1.00
6	Ceph-Phen-Rif-Tet-Mac-Lac	1	0.5	
7	Ami-Sul-Car-Ceph-Rif-Tet-Mac	1	0.5	
8	Ami-Flu-Sul-Ceph-Phe-Rif-Tet-Mac	1	0.6	
	*V. parahaemolyticus*			
6	Ami-Car-Ceph-Rif-Tet-Mac	1	0.5	1.00
	Sul-Car-Ceph-Rif-Tet-Mac	1	0.5	
	*V. algivorus*			
4	Mac-Tet-Ceph-Flu	1	0.3	0.83
6	Ami-Sul-Car-Ceph-Phe-Tet	1	0.5	
7	Ami-Flu-Sul-Ceph-Rif-Tet-Mac	1	0.6	
	Car-Ceph-Phe-Rif-Tet-Mac-Lac	1	0.5	
9	Ami-Flu-Sul-Car-Ceph-Phe-Rif-Tet-Lac	2	0.7	
	*V. fortis*			
5	Phe-Rif-Tet-Mac-Lac	1	0.5	1.00
6	Ami-Sul-Car-Ceph-Tet-Mac	1	0.5	
	Ami-Sul-Car-Rif-Tet-Mac	1	0.6	
7	Ami-Sul-Car-Ceph-Rif-Tet-Lac	1	0.6	
	Car-Ceph-Phe-Rif-Tet-Mac-Lac	1	0.5	
8	Ami-Flu-Sul-Car-Rif-Tet-Mac-Lac	1	0.6	
	*V. panuliri*			
5	Ami-Car-Tet-Mac-Lac	1	0.3	1.00
	Car-Ceph-Rif-Tet-Mac	1	0.4	
6	Ami-Sul-Rif-Tet-Mac-Lac	1	0.5	
	*V. stylophorae*			
4	Ami-Car-Tet-Mac	1	0.3	1.00
6	Sul-Car-Ceph-Rif-Tet-Mac	1	0.4	

## Discussion

Water is a critical aspect of sustainable development, and human beings play an important role in eliminating poverty and health difficulties. As a result, the production of wastewater is an ongoing process around the world. Hospitals are significant institutions that generate wastewater that may contain contaminants such as pathogens as well as antibiotic-resistant strains; the discharge of this wastewater without first undergoing treatment may contribute to difficulties in maintaining public health. This study examined the distribution of *Vibrio* species in State Specialist Hospital wastewater as part of efforts to provide information for the control of the spread of infectious diseases. The study revealed the presence of *Vibrio spp* (29; 58%) in the wastewater of the hospital. Other investigators in and out of Nigeria have also reported the presence of *Vibrio spp* in hospital wastewater [26,27]. Hospital wastewater had been reported to be implicated in the dissemination of pollutants which includes pathogenic bacteria as well as genes that could enhance the pathogenicity of these bacteria [19]. Six different species of *Vibrio* were identified by sequencing of which 17.4% were *V. cholerae*. The incidence of *Vibrio cholerae* in this study agrees with the findings of Mustapha and Imir, who also recovered *V. cholerae* from hospital wastewater in Nigeria [27]. *Vibrio cholerae* is the causative agent of the common form of *Vibrio* pathology and leads to cholera via the secretion of enterotoxins, which causes flushing of important nutrients in the cell, like sodium and water, leading to stooling and serious dehydration. Sub-Saharan Africa had been widely involved in several epidemics of cholera [28], where the risk associated with cholera infection is high. One of the ways through which *V. cholerae* enters the water bodies is through the discharge of untreated or partially treated wastewater into this environment and hospital wastewater is a key player in this scenario [3]. *Vibrio parahaemolyticus* is another species found in this study that had also been regarded as a human pathogen. *V. parahaemolyticus* is a halophile that has been linked to the onset of gastroenteritis in countries all over the world, including Nigeria [29].

It had been proven by many scientists that this human pathogen occurs in many geographical areas and its infection in many cases is linked to the consumption of poorly prepared local meals [3]. This pathogen is known to cause three main infections which are gastroenteritis, wound infection, and septicemia with gastroenteritis being the most common and is characterized by several symptoms (such as watery and/or blood attained diarrhea associated with some pains, nausea, vomiting, headache, and fever) [3,30]. The presence of this pathogen in State specialist hospital Okitipupa´s wastewater poses a public health threat due to its link with sea or marine food-borne infectious diseases that had also been reported in other countries [21]. This wastewater could gain access to the nearby water bodies thereby contaminating the water as well as the aquatic animals which will be transmitted to humans via consumption of the contaminated seafood. Other *Vibrio* species that were isolated in this study included *Vibrio algivorus, Vibrio fortis, Vibrio panuliri*, and *Vibrio stylophorae. Vibrio algivorus*, first isolated from the gut of a turban shell marine snail in Japan was proposed by Doi *et al*. to be a novel strain with the ability to degrade and/or metabolize alginate as well as agarose-assimilator which could be utilized in bio-fermentation [31]. Hence, the high incidence of this species (26.1%) in the wastewater samples as observed in this study could be a pointer to novel strains of *Vibrio* in the study area. *Vibrio fortis* (26.1%), another *Vibrio* species isolated in this study had been recovered from spotted rose snapper and from crown-of-thorns starfish in Australia and Guam [32,33]. It was also found to be one of the most common *Vibrio* species in Venezuela's Cariaco Basin [34]. This organism is a pathogenic species that has been linked to infectious diseases in many aquatic animals, implying that if wastewater is dumped into bodies of water or mistakenly enters the aquaculture environment, farmers may suffer economic losses [35]. The incidence of *Vibrio panuliri* in this study could be a pointer to some unknown factors in the environment because this organism was previously isolated from eggs of spiny lobster in the Andaman Sea [36]. *Vibrio stylophorae* is a potential pathogen that is indigenous to the estuarine and marine environment. It has been linked to seafood-borne gastroenteritis globally via the consumption of shellfish that are not properly processed [37]. Their presence could be via faecal contamination of shellfish that grows in that environment, handlers of food, and unhygienic storage condition. The outbreak of *Vibrio stylophorae* gastroenteritis has received considerably less attention worldwide, especially in countries near coastal waters. Okereke and Anyiam reported that re-contamination of cooked seafood held at high temperatures is possible allowing rapid growth which could be visible through the high number of *V. stylophorae* [38]. The incidence of diverse species of *Vibrio* in the wastewater samples collected from the State hospital could be a reflection of the water source before use, the storage container, or probably a contaminated environment.

Antibiotic resistance in bacteria remains a global threat to the application of chemotherapeutic agents and thus increases the rate of death via infectious diseases. It is of great importance to frequently assess the susceptibility of potentially pathogenic bacteria to commonly used antibacterial agents since the majority of the resistance determinants in these organisms can be transferred from one bacterium to another via mobile genetic elements. This study also determined the antibiotic susceptibility profile of the *Vibrio* species isolated from hospital wastewater samples. About average of the isolated *Vibrio* species was susceptible to ciprofloxacin, cotrimoxazole, and chloramphenicol. *Vibrio cholerae* (n=4) were 100% susceptible to gentamicin and cotrimoxazole and 75% susceptible to amikacin, ciprofloxacin and ampicillin, and 50% susceptible to trimethoprim, streptomycin, and chloramphenicol. Susceptibility to appreciable numbers of antibiotics found in this study agrees with the findings of other researchers [13,21]. Resistance to streptomycin, as well as chloramphenicol observed in *V. cholerae*, is in agreement with the result of Okoh and Igbinosa which was attributed to the rapidity in the distribution of these resistance determinants in *Vibrio* species as a result of a novel type of conjugative transposon [39]. High resistance to cefotaxime (100%) and tetracycline (75%) in *V. cholerae* isolated is in accordance with the result of Adesiyan *et al*. and Ottaviani *et al*. [21,40]. All the *Vibrio parahaemolyticus* (n=2) isolates were susceptible to amikacin, ciprofloxacin, cotrimoxazole and chloramphenicol. They were however commonly resistant (50 - 100%) to erythromycin, rifampicin, amoxicillin and meropenem, which is in accordance with other findings and this could be as a result of the transfer of antibiotic resistance determinant from one species of bacteria to another [21]. Previous studies have shown that wastewater from diverse sources plays an important role in the transmission of antibiotic resistance determinants in aquatic environments. An appreciable amount of antibiotics used in clinical settings for the treatment of infections in humans is discharged in a biological mode via urination and feaces. Most of this wastewater is usually discharged into the septic tanks and/or treatment plants which are released as inappropriately treated effluents into environments [41,42].

*Vibrio fortis* had been previously identified as a potential pathogen to aquatic animals due to its occurrence in healthy and diseased lion´s paw scallop larvae, diseased *C. gigas* larvae, shrimp larvae, Atlantic salmon, as well as seawater [43]. Wang *et al*. also reported high pathogenicity of *V. fortis* in their seahorses´ experiment where they recorded 100% death in the infected animals [35]. The high resistance displayed by *V. fortis* to the antibiotics tested in this study calls for concern because contamination of aquaculture systems with this type of wastewater would result in economic loss and may have adverse effects on humans. To the best of our knowledge, this is the first report of the occurrence of *V. fortis* in wastewater in Nigeria. All the *Vibrio* species isolated in this study differ in their reactions to antibiotics despite some similarities regarding species identity except two *V. algivorus* which showed resistance to nine classes of antibiotics with the same phenotypes. *Vibrio algivorus* was first proposed by Doi *et al*. [44] when isolated from the gut of a turban shell sea snail in Japan and contrary to their report the species were susceptible to vibriostatic agents, the strains of *V. algivorus* in our study resisted (resistance ranges between 33.3 - 83%) the majority of the tested antibiotics. This finding had been previously reported by Igbinosa [5]. Although, little is known about the significance of this organism, its occurrence in the present study showed that the environment could be a reservoir for novel strains which could be of medical or industrial importance. *Vibrio panuliri* was first isolated from a spiny lobster in India. This organism was resistant to some antibiotics that had been reported to be susceptible to by other investigators [36] and such antibiotics included rifamycin and tetracycline meanwhile the result aligns with the previous resistance to erythromycin and gentamicin. *V. stylophorae* had been previously reported to be associated with gastroenteritis and those isolated in this study were resistant to commonly used antibiotics which could lead to the non-responsiveness of associated infections to therapy. Contrary to the previous report of high resistance of *Vibrio vulnificus* (41%) and *V. cholerae* (50%) isolated from water resources in southwest Nigeria to ampicillin [21], resistance to ampicillin by *Vibrio* species in this study was less than 35%. This could be attributed to the diversity in the environment in relation to activities.

All the *Vibrio* species showed multiple antibiotic resistance phenotypes which range between 4-9 classes of antibiotics. Indiscriminate use of antibiotics in diverse sectors is an important contributor to the emergence of antibiotic resistance thereby affecting the efficacy of antibiotics [21]. This calls for effective monitoring and management of this class of antibiotics in aquaculture and healthcare to reduce public health risks. Multiple Antibiotics Resistant index of all the isolates were higher than the 0.2 recommended standard which indicates that the source of the wastewater is a highly antimicrobial-contaminated area. The high MAR index (0.3 - 0.7) observed in *Vibrio* species isolated in this study corresponds to the findings of other researchers [21,45] but differs from the values recorded (0.00 - 0.22) in *V. parahaemolyticus* isolated from a molluscan fish farm on the Korean coast by Mok *et al*. [46]. The high MAR indices as observed in this study is not surprising as the activities commonly carried out in the environment deals with the treatment of infections which require the use of antibiotics in the institution. The disposal or the practice of burying wastewater containing multiple antibiotics resistant pathogenic bacteria such as *Vibrio* strains is a serious public health concern in both human and veterinary medicine, especially in the study area which requires the immediate attention of the concerned bodies. The discrepancy in the MAR index observed among the six *Vibrio* species in this study could be due to the variation in the strains thereby having different pressures for antibiotic resistance at varied proportions.

**Limitations:** the limitation of our study is the small number of isolates we analyzed due to lack of funds; larger studies are needed to further investigate the magnitude of the occurrence of resistant pathogens in hospital wastewater in this environment.

## Conclusion

This study has revealed the presence of diverse multiple antibiotic-resistant *Vibrio spp* in wastewater from Okitipupa State Specialist Hospital, Ondo State, Southwest Nigeria with *V. algivorus* and *V. fortis* having the highest prevalence rate. Emerging pathogens like some species of *Vibrio* are of great importance as a result of their public health and economic-related consequences. Wastewater management including direct analysis of certain pathogenic bacteria of public and environmental health concern is of essence especially in the study area due to their high consumption rate of aquatic animals as well as daily contact with water bodies. High prevalence of multiple antibiotic-resistant *Vibrio* species in untreated hospital wastewater effluent indicates their ability to persist in the hospital environment. Hence, it is of great importance to develop liquid waste treatment plant with adequate chlorination so as to inactivate pathogens in treated wastewater effluents to the nearest minimum before being discharged into the environment.

### What is known about this topic

- *Hospital wastewater has been reported to be implicated in the dissemination of pollutants which include pathogenic bacteria;*
- *Although the incidence of Vibrio cholerae has been determined in hospital effluents, there is a dearth of information regarding the occurrence of other Vibrio species*.

### What this study adds

- *The study provided information on the distribution of Vibrio cholerae and other species in hospital wastewater by sequencing;*
- *The study also provided information on the susceptibility profile of Vibrio spp in hospital wastewater;*
- *This is the first report of the occurrence of Vibrio fortis in wastewater in Nigeria*.
